# An audit of platelet transfusions at a tertiary care center: New opportunities for patient blood management with the 2025 AABB/ICTMG platelet guidelines

**DOI:** 10.1111/trf.70052

**Published:** 2026-01-12

**Authors:** Rylee Yakymi, Claudia S. Cohn

**Affiliations:** ^1^ Department of Laboratory Medicine and Pathology University of Minnesota Minneapolis Minnesota USA

**Keywords:** audits, patient blood management, platelet, transfusions

## Abstract

**Background:**

Platelet transfusions are an important tool to prevent and stop bleeding. Thresholds for pretransfusion platelet counts have been studied in various patient populations, yielding evidence‐based guidelines. The Association for the Advancement of Blood and Biotherapies (AABB) collaborated with the International Collaboration for Transfusion Medicine Guidelines (ICTMG) to develop a platelet guideline with new and updated recommendations for different patient populations. The goal of this study was to determine platelet transfusion appropriateness in a large tertiary care hospital, identify common scenarios with deviations from guidelines, and assess the effect that the new AABB/ICTMG guidelines could have on platelet utilization.

**Study Design and Methods:**

A retrospective 8‐week audit of platelet transfusions at a university hospital was conducted using institution‐specific adjudication criteria. A second audit applied the AABB/ICTMG recommendations. Patient demographics, laboratory values, and transfusion details were collected with an electronic audit tool. Each platelet (PLT) order was adjudicated through manual record review.

**Results:**

A total of 1667 units of apheresis PLT were transfused to 312 patients. Using current hospital guidelines, 163 of 1288 adult (12.7%) and 44 of 379 pediatric orders (11.6%) were deemed inappropriate and 119 adult (9.2%) and 24 pediatric (6.3%) orders were indeterminate. The second audit, which applied recommendations from the 2025 AABB/ICTMG platelet guideline, found multiple PLT transfusions that would be newly noncompliant.

**Discussion:**

There is an incongruency between clinical practice across various specialties and evidence‐based platelet guidelines for platelet transfusions. The new AABB/ICTMG guidelines create an opportunity to reduce unnecessary platelet transfusions in several patient populations.

**Abbreviations**
AABB: Association for the Advancement of Blood and Biotherapies
ICTMG: International Collaboration for Transfusion Medicine Guidelines
PLT: platelet
RBC: red blood cell
U.S.: United States
GRADE: Grading of Recommendations, Assessment, Development, and Evaluation
UMMC: M‐Health Fairview University of Minnesota Medical Center
OR: operating room
CPB: cardiopulmonary bypass
IR: interventional radiology
BMT: bone marrow transplant
ICU: intensive care unit
ECMO: extracorporeal membranous oxygenation

## 1 INTRODUCTION

Patient blood management efforts have helped reduce red blood cell (RBC) transfusions in the United States by >13.9% since 2008,^1^, ^2^ yet similar efforts for platelet transfusions have not been widespread despite an increase in demand for platelet transfusion in the United States^3^, ^4^, ^5^, ^6^ and other high‐resource countries as well.^7^ The Association for the Advancement of Blood and Biotherapies (AABB) has used systematic reviews and Grading of Recommendations, Assessment, Development, and Evaluation (GRADE) methodology to develop recommendations for appropriate platelet transfusions in several patient populations;^8^ the AABB's first platelet guideline was published in 2015.^9^

Our institution used the electronic medical record and laboratory information system to create a daily report of transfusions with relevant laboratory values from before and after each transfusion. These assembled data were used to regularly audit RBC transfusions, applying the recommendations from published guidelines.^10^, ^11^, ^12^ The results of these audits were used to educate clinical teams about appropriate transfusion practice. This effort led to a 15% reduction in RBC use at our hospital in 2013^13^ that has been sustained with spot audits, best practice alerts and general education.

A recent joint AABB/ICTMG expert panel used a systematic review and meta‐analysis to develop an update to the 2015 AABB platelet guidelines.^8^ This guideline includes several new and/or updated recommendations for appropriate evidence‐based platelet transfusions. These new guidelines were applied in a reassessment of our hospital's platelet transfusion practice. Firstly, the hospital's current guidelines were used to audit platelet transfusions ordered over an 8‐week period. These transfusions were then subjected to a second audit that applied the new guidelines. This approach allowed us to identify new opportunities to expand pharmacy benefit management (PBM) efforts and reduce inappropriate platelet transfusions.

## 2 MATERIALS AND METHODS

### 2.1 Patient eligibility

Retrospective 8‐week audits (May and June 2024) of all platelet transfusion orders occurring at M‐Health Fairview University of Minnesota Medical Center (UMMC) were conducted. The audits were overseen by a medical student, transfusion medicine physician, and a transfusion safety officer. Institutional review board approval was obtained.

### 2.2 Transfusion events

Each unit transfused was considered a separate event. In addition, when patients received more than one platelet unit in a calendar day, the audit focused on the first transfusion. Pediatric patients usually have apheresis platelet units split based on patient weight per hospital policy.

### 2.3 Data collection

Daily transfusion data were extracted from the electronic medical record and laboratory information system into an Excel spreadsheet. Demographic data included patient age, sex, and birth date. Clinical data included treatment department and service, ordering clinician, and treatment indication. Transfusion of each blood component was integrated with pertinent pre‐ and posttransfusion laboratory values, such as platelet counts, hemoglobin/hematocrit, INR, and fibrinogen.

### 2.4 Inclusion and exclusion criteria

All adult and pediatric patients who received a platelet transfusion were included in the data set. As most guideline recommendations are specific for stable, non‐bleeding patients, the current hospital guideline provide recommendations for patients in the perioperative setting, but does not provide specific recommendations for intraoperative transfusions; thus all operating room (OR) transfusions were excluded from the original audit. However, the 2025 AABB platelet guideline includes a recommendation for cardiac surgery patients undergoing cardiopulmonary bypass (CPB) procedures. As a result, patients undergoing CPB were included in the second audit that applies AABB/ICTMG guidelines.

### 2.5 Assessing appropriateness criteria

A medical trainee audited each platelet transfusion by applying criteria as described below. The audits were checked by the attending physician and/or the Transfusion Safety Officer. No blinding occurred. Each transfusion was adjudicated as either appropriate, inappropriate, or indeterminate (due to insufficient data or absence of pre‐platelet counts). The primary audit used our hospital's guidelines for appropriate platelet transfusions to adjudicate pediatric and adult platelet transfusions. The hospital guidelines were developed from a variety of published sources, but most are based on the AABB 2015 platelet guideline^9^ (Adjudication criteria using current hospital guidelines are shown in Table 1). The second audit applied most of the 11 recommendations from the 2025 AABB/ICTMG guidelines (see Table 4). The number of RBC units transfused served as a surrogate measure of bleeding for some of the 2025 recommendations. In our facility, patients undergoing CPB receive an average of 2 RBC units in a procedure; therefore, we considered any patient who received less than 2 RBC units to be without major hemorrhage.

**TABLE 1:** Current hospital guidelines for platelet transfusions.

Patient clinical indications	Appropriate platelet count (×10^9^/L)	Inappropriate platelet count (×10^9^/L)
Baseline for stable adult patient	≤10	≥15^a^
Thrombocytopenia due to hematologic malignancies, hematopoietic cell transplant, cytotoxic chemotherapy, or sepsis		
Mucosal bleeding	≤20	>20
Central venous catheter placement		
Low‐risk diagnostic endoscopic procedures		
Bronchoscopy with bronchoalveolar lavage		
Lumbar punctures in patients *with* hematologic malignancies		
Bone marrow aspiration/biopsy		
Acute promyelocytic leukemia	≤30	>30
Patients taking defibrotide		
Active bleeding	≤50	>50
Surgery or other invasive procedures (+1 week grace period for postoperative healing)		
Lumbar punctures		
Therapeutic endoscopic procedures		
Neuraxial endoscopic procedures		
Coagulopathy, including DIC, with bleeding		
Severe infection AND bleeding		
Patients with sickle cell anemia or with inborn errors of metabolism immediately following marrow transplant		
Infant <1 month of age		
Neuro‐ or ophthalmologic surgery	≤100	>100
Massive Blood Transfusion Protocol/hemorrhagic blood loss.	No specific threshold; deemed appropriate based on clinical assessment of MTP activating physician.	

^a^ Transfusion qualification buffer given with the understanding that clinical assessment is likely more involved at this restrictive baseline threshold.

### 2.6 Statistical analysis

All analyses were conducted with Microsoft Excel (Excel v16.87; Microsoft Corp.; Albuquerque, NM, USA). Variables analyzed included determining appropriateness by treatment department and pre‐PLT count. Categorical variables were summarized as frequencies and percentages.

## 3 RESULTS

### 3.1 Platelets transfused and indications

A total of 1667 units of apheresis PLT were transfused to 312 patients. Adult and pediatric patient demographics, laboratory, and transfusion data are shown in Table 2. Adult platelet transfusions occurred most often in the setting of bone marrow transplant (BMT) (33.2%), hematology‐oncology (23.1%), general medicine (15.9%), and the operating room (15.3%). The most common pediatric PLT transfusion settings were pediatric general medicine (56.5%), BMT (23.5%), and neonatal intensive care unit (NICU) (12.7%). Prophylactic transfusion for non‐bleeding patients with hypoproliferative thrombocytopenia predominated at 54.0% of all adult transfusions in this audit. Other common indications were therapeutic transfusions for extracorporeal membrane oxygenation (ECMO) and CPB and liver transplantation, collectively encompassing 20% of all transfusions between adult and pediatric patients. Of the 1288 adult orders, pretransfusion platelet counts were available in 732 orders (90.8%). Of the 379 pediatric orders, pretransfusion counts were available for 351 (92.6%).

**TABLE 2:** Patient demographics and transfusion details.

Number of platelet orders	ADULT (*n* = 1288 orders)	PEDIATRIC (*n* = 379 orders)
Sex at birth		
Male	685 (53.2%)	275 (72.6%)
Female	603 (46.8%)	104 (27.4%)
Age		
Pediatric: ≤7 days	‐	11 (2.9%)
Pediatric: >8 days–6 years	‐	215 (56.7%)
Pediatric: 7–12 years	‐	141 (37.2%)
Pediatric: 13–17 years	‐	12 (3.2%)
Adult: 18–35 y	284 (22.0%)	‐
Adult: 36–65 y	596 (46.3%)	‐
Adult 66–80 years	375 (29.1%)	‐
Adult: >80 years	33 (2.6%)	‐
Pretransfusion PLT count (×10^9^/L)		
≤10	302 (23.4%)	59 (15.6%)
11–20	354 (27.5%)	73 (19.3%)
21–30	144 (11.2%)	74 (19.5%)
31–50	200 (15.5%)	87 (23.0%)
51–100	122 (9.5%)	43 (11.3%)
>100	47 (3.7%)	15 (3.9%)
No count	119 (9.2%)	28 (7.4%)
Ordering provider specialty	428 (33.2%)	89 (23.5%)
BMT	298 (23.1%)	2 (0.5%)
Hematology‐Oncology	205 (15.9%)	214 (56.5%)
General Medicine/Pediatrics	197 (15.3%)	1 (0.3%)
Surgery and Solid Organ Transplant	84 (6.5%)	2 (0.5%)
Cardiology	26 (2.0%)	‐
Medical ICU	9 (0.6%)	‐
Cardiac ICU	5 (0.4%)	‐
Emergency Medicine	‐	48 (12.7%)
Neonatology	36 (3.0%)	23 (6.0%)
Other		
Transfusion indication		
Hypoproliferative thrombocytopenia w/o bleeding	695 (54.0%)	240 (63.3%)
Hypoproliferative thrombocytopenia w/ active bleeding	182 (14.1%)	59 (15.5%)
CPB and ECMO	192 (14.9%)	56 (14.8%)
Perioperative	147 (11.4%)	12 (3.2%)
Pre‐procedural	65 (5.1%)	12 (3.2%)
Other	7 (0.5%)	‐

Abbreviations: BMT, bone marrow transplant; CPB, cardiopulmonary bypass; ECMO, extracorporeal membranous oxygenation; ICU, intensive care unit.

### 3.2 Appropriateness of adult PLT transfusions

**FIGURE 1:**
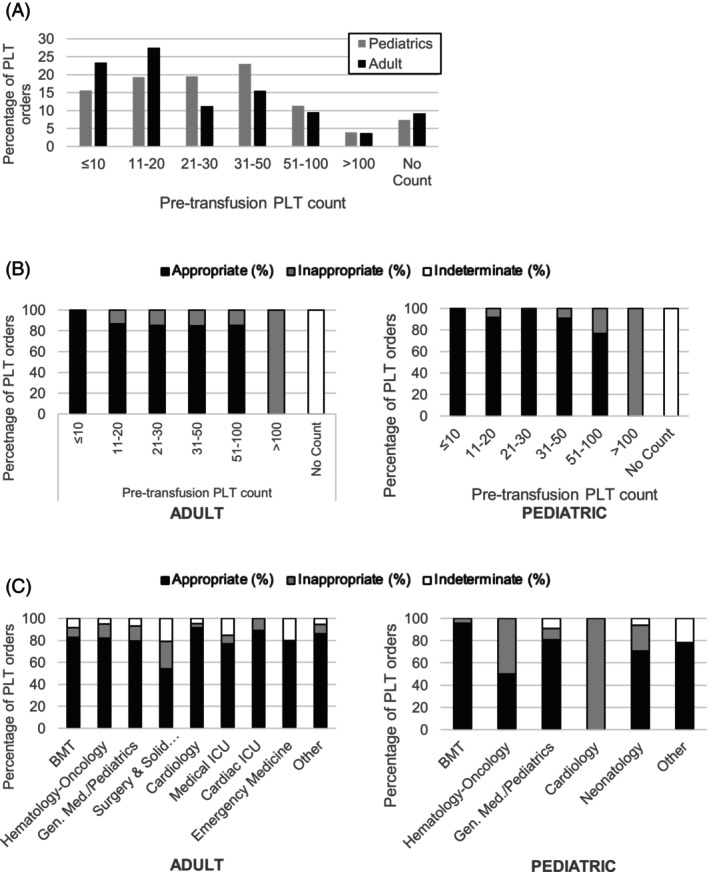
Summary of platelet audit details for all pediatric (including neonates) and adult patients. Pretransfusion platelet counts compared between (A) adult and pediatric orders, (B) overall appropriateness compared by pretransfusion platelet count (adult and pediatric patients), and (C) overall appropriateness compared between treatment departments (adult and pediatric patients).

Table 3 shows that 1008 (78.3%) of the adult platelet transfusions were deemed appropriate, 163 (12.7%) inappropriate, and 117 (9.1%) indeterminate. There were 70 patients who received more than one platelet unit in a single day, and of those, 32 experienced more than 1 day in which >1 unit was transfused. When >1 unit was given in a day, the first transfusion was appropriate in 93% (128/137) of cases, while 5% (7/137) of first transfusions were either inappropriate or indeterminate. Nearly half of the ordered platelet transfusions had PLT pre‐counts of either ≤10 × 10^9^/L (23.4%) or 11–20 × 10^9^/L (27.5%) (Figure 1A). Higher PLT pre‐counts were more frequently rated as inappropriate; 14.8% for 51–100 × 10^9^/L and 100% for >100 × 10^9^/L (Figure 1B). Of all the treatment departments included in this audit, surgery (perioperative; 24.9%), general medicine (13.7%), and hematology‐oncology (13.1%) had the highest rates of inappropriate PLT transfusion orders (Figure 1C). In evaluating indications for PLT orders, ECMO was the most common cause of inappropriate transfusions in the first audit.

**TABLE 3:** Audit using current hospital recommendations: Adjudication of platelet orders by transfusion details.

Pre‐PLT count	ADULT (*n* = 1288 orders)			PEDIATRIC (*n* = 379 orders)		
	Appropriate	Inappropriate	Indeterminate	Appropriate	Inappropriate	Indeterminate
≤10	302 (100%)	0	‐	59 (100%)	0	‐
11–20	307 (86.7%)	47 (13.3%)	‐	67 (91.8%)	6 (8.2%)	‐
21–30	123 (85.4%)	21 (14.6%)	‐	73 (98.6%)	1 (1.4%)	‐
31–50	170 (85.0%)	30 (15.0%)	‐	79 (90.8%)	8 (9.2%)	‐
51–100	104 (85.2%)	18 (14.8%)	‐	33 (76.7%)	10 (23.3%)	‐
>100	0	47 (100%)	‐	0	15 (100%)	‐
No count	‐	‐	119 (100%)	‐	‐	28 (100%)
Ordering provider specialty						
BMT	354 (82.7%)	38 (8.9%)	36 (8.4%)	85 (95.5%)	4 (4.5%)	0
Hematology‐Oncology	244 (81.9%)	39 (13.1%)	15 (5.0%)	1 (50.0%)	1 (50.0%)	0
General Medicine/Pediatrics	163 (79.5%)	28 (13.7%)	14 (6.8%)	173 (80.8%)	22 (10.3%)	19 (8.9%)
Surgery and Solid Organ Transplant	107 (54.3%)	49 (24.9%)	41 (20.8%)	‐	‐	‐
Cardiology	77 (91.7%)	3 (3.5%)	4 (4.8%)	0	2 (100%)	0
Medical ICU	20 (76.9%)	2 (7.7%)	4 (15.4%)	‐	‐	‐
Cardiac ICU	8 (88.9%)	1 (11.1%)	0	‐	‐	‐
Emergency Medicine	4 (80%)	0	1 (20%)	‐	‐	‐
Neonatology	‐	‐	‐	34 (70.8%)	11 (22.9%)	3 (6.3%)
Other	31 (86.1%)	3 (8.3%)	2 (5.6%)	18 (78.3%)	0	5 (21.7%)
Transfusion indication						
Hypoproliferative thrombocytopenia w/o bleeding	570 (82.0%)	88 (12.7%)	37 (5.3%)	204 (85.0%)	18 (7.5%)	18 (7.5%)
Hypoproliferative thrombocytopenia w/ active bleeding	155 (85.2%)	11 (6.0%)	16 (8.8%)	53 (89.8%)	4 (6.8%)	2 (3.4%)
CPB and ECMO	126 (65.6%)	44 (22.9%)	22 (11.5%)	40 (71.4%)	10 (17.9%)	6 (10.7%)
Perioperative	101 (68.7%)	11 (7.5%)	35 (23.8%)	8 (66.7%)	3 (25.0%)	1 (8.3%)
Pre‐procedural	53 (81.6%)	6 (9.2%)	6 (9.2%)	6 (50.0%)	5 (41.7%)	1 (8.3%)
Other	3 (42.8%)	3 (42.8%)	1 (14.4%)	‐	‐	‐

Abbreviations: BMT, bone marrow transplant; CPB, cardiopulmonary bypass; ECMO, extracorporeal membranous oxygenation; ICU, intensive care unit.

### 3.3 Appropriateness of pediatric PLT transfusions

A total of 311 (82.1%) pediatric orders were deemed appropriate, 40 (10.6%) inappropriate, and 28 (7.3%) indeterminate. Of all the transfusions, there was a fairly even distribution given for each PLT pre‐counts category <100 × 10^9^/L (Figure 1A). Unlike the adults, inappropriate pediatric orders also had approximately equal occurrences across all pre‐count levels, except for >100 × 10^9^/L with 15 incidences (Figure 1B). Inappropriate PLT orders occurred more frequently in the setting of NICU (22.9%) for indications of ECMO and hypoproliferative thrombocytopenia without bleeding (Figure 1C). There were 11 patients who received more than one platelet unit in a single day, and of those, 9 experienced more than 1 day when >1 unit was transfused. When >1 unit was given in a day, the first transfusion was appropriate in 100% (75/75) of cases.

### 3.4 A comparison of audit results using current hospital guidelines versus 2025 AABB/ICTMG platelet guidelines

The recent publication of the 2025 AABB/ICTMG platelet guidelines prompted a new audit of the 1667 transfusions based on the 11 recommendations developed by the guideline panel (see Table 4); however, some recommendations were not included in this exercise. Specifically, there were 4 new recommendations that were similar to the hospital's current guidelines, and 1 recommendation that applied to patients with dengue, which is not usually seen in the hospital's patient population. Also, there were new recommendations for low‐ versus high‐risk interventional radiology (IR) procedures, but our hospital currently lacks a risk stratification system for IR. Finally, a new recommendation is for patients undergoing cardiac surgery, including those using CPB, but the current hospital guidelines do not regulate intraoperative transfusions. Nonetheless, patients on CPB were included in the second audit, albeit without a comparator group. Thus, 5 recommendations were included in the second audit, which demonstrated shifts from compliance to noncompliance for patient populations that included: neonates; lumbar punctures; cardiac surgery; and central venous catheter placements among others. Overall, 64% of the transfusions found appropriate with current hospital guidelines were now inappropriate with the new recommendations. Patients undergoing cardiac surgery with CPB without major hemorrhage had 56/80 (70%) adult and pediatric transfusions that would have been noncompliant with the AABB guideline. In addition, 9 of 23 (39.1%) and 9 of 13 (69.2%) transfusions for lumbar punctures and central venous catheters, respectively, shifted from appropriate to inappropriate as well.

**TABLE 4:** Comparison of audits adjudicated with current hospital guidelines versus AABB/ICTMG recommendations for platelet transfusions, using 2025 Guideline.^8^

	Patient population	Current hospital guidelines				AABB/ICTMG recommendations			
	Within guidelines PLT transfusion?		Yes	No	Ind		Yes	No	Ind
1	Hypoproliferative thrombocytopenia in non‐bleeding patients receiving chemotherapy or undergoing allogeneic stem cell transplant	<10 × 10e3/uL	N/A			<10 × 10e3/uL	N/A		
2	Consumptive thrombocytopenia in neonates without major bleeding	**<50 × 10e3/uL**	**5**	**4**	**0**	<25 × 10e3/uL	**5**	**4**	**0**
3	Patients undergoing lumbar puncture	**< 50 × 10e3/uL**	**14**	**5**	**4**	<20 × 10e3/uL	**5**	**14**	**4**
4	Patients with consumptive thrombocytopenia due to Dengue without major bleeding	No guidance	N/A			No platelet transfusion	N/A		
5	Hypoproliferative thrombocytopenia in non‐bleeding adults undergoing autologous stem cell transplant or with aplastic anemia	<10 × 10e3/uL	2	1	0	Prophylactic platelet transfusion is not recommended	1	2	0
6	Adults with consumptive thrombocytopenia without major bleeding	<10 × 10e3/uL	N/A			<10 × 10e3/uL	N/A		
7	Adults undergoing central venous catheter placement in compressible anatomic sites	< 50 × 10e3/uL	11	1	1	<10 × 10e3/uL	2	10	1
8	Adults undergoing interventional radiology procedure: Low risk/High risk	<50 × 10e3/uL (Low and high risk)	N/A			<20 × 10e3/uL (Low) < 50 × 10e3/uL (High)	N/A		
9	Adults undergoing major nonneuraxial surgery	<50 × 10e3/uL	N/A			<50 × 10e3/uL	N/A		
10	Patients without thrombocytopenia undergoing cardiovascular surgery in the absence of major hemorrhage, including those receiving cardiopulmonary bypass	No guidance for intraoperative transfusions	N/A			No platelet transfusion	24	56	0
11	Nonoperative intracranial hemorrhage in adults with platelet count greater than 100 × 10^3^/μL, including those receiving antiplatelet agents	<100 × 10e3/uL	32	0	4	No platelet transfusion	28	4	4

## 4 DISCUSSION

We found that 78.3% of platelet transfusions in the adult population and 82.1% in the pediatric population were appropriate based on our current hospital guidelines. Compliance with guidelines differed among the hospital services, with most services, including BMT and cardiology, having >80% of platelet transfusions compliant with hospital guidelines. Conversely, surgery, general medicine, and hematology‐oncology had the highest rates of noncompliance. In the pediatric population, BMT had >90% appropriate platelet transfusions, whereas the NICU and “Other” had the lowest rates of compliance with hospital guidelines. Platelet transfusions for ECMO patients were often deemed inappropriate, but it is difficult to distinguish when ECMO patients are bleeding, and platelet transfusions for bleeding patients are considered appropriate. Thus, findings for this patient population remain uncertain and were not reported in this study.

The overall rate of appropriate transfusions found at our hospital compares well with other reports in the literature. Audits have shown a wide range of compliance with local or regional guidelines, with high resource countries reporting 47%–89% of transfusions aligned with guidelines^14^, ^15^, ^16^, ^17^, ^18^, ^19^, ^20^, ^21^, ^22^, ^23^, ^24^, ^25^ while lower‐middle income countries reported 19%–94% appropriate PLT transfusions.^26^, ^27^, ^28^, ^29^, ^30^ When PLT transfusions were parsed by specialty, the intensive care unit^20^ and surgical specialties^16^, ^21^, ^22^, ^27^ had the lowest rates of compliance while pediatric services had comparatively better rates of compliance than adult services.^23^ Some groups have tried to improve compliance using prospective review^31^, ^32^, ^33^ or clinical decision support tools with mixed results.^34^, ^35^

The 2025 AABB/ICTMG guidelines provided several new and updated recommendations, and it is not surprising that our second audit found clusters of noncompliance. The recommendation against platelet transfusions for cardiac surgery patients without major hemorrhage caused the most reversals in audit results. The current hospital policy is to consider all OR transfusions as appropriate, since patients are “open” and decisions to transfuse during a procedure often depend upon visualization of the surgical field rather than a platelet count. However, the general practice of transfusing a unit of platelets immediately after moving the patient off‐pump is not compliant with the 2025 recommendation. Other new recommendations, such as using a threshold of <10 × 10^9^/L for central venous catheter placement in compressible anatomic sites, will require extensive education before new clinical practice patterns take hold. The new guideline for IR has the potential to change practice patterns in low‐risk IR procedures, but defining low‐ versus high‐risk IR procedures will require close cooperation between interventional radiologists and transfusion medicine. In countries with a national blood policy, the risk stratification will be developed with experts in these fields. In the United States, however, each hospital will make its own decisions, hopefully guided by relevant societies.

This study has several important limitations. Retrospective audits largely depend on lab values that do not fully reflect the clinical status of the patient. It is standard practice to use recommended platelet count thresholds to guide platelet orders; however, it is also standard to note that lab values alone should not guide transfusion decisions. Yet, this is all that is available for most audits. The audit could be improved by incorporating data from thromboelastograms, which are sometimes used in the hospital's intensive care unit (ICU) and operating rooms. Also, the second audit with the AABB/ICTMG guidelines is based on upcoming changes in the hospital; therefore, the findings of “noncompliance” reflect potential changes for practice rather than the true state of platelet transfusions in the hospital. Finally, there are still settings in which there is no specific guidance provided on platelet transfusion appropriateness, such as chronic hypoproliferative thrombocytopenia not undergoing chemotherapy or allogeneic stem cell transplant. This limits the potential of audits overall.

The strength of this study lies mainly in the opportunities revealed by applying the 2025 AABB/ICTMG recommendations to a retrospective audit. While our findings apply directly to our hospital, it is likely that other sites will also need to update their hospital guidelines to reflect the new recommendations.

The audits in this study have demonstrated the potential effect the new AABB/ICTMG guidelines could have on platelet use in large tertiary centers. The first audit, which used hospital guidelines largely based on the 2015 AABB guideline, set the baseline for current practice. The second audit, which used the new AABB/ICTMG recommendations, demonstrated the potential changes that could come about with adherence to the new guidelines. While some of the findings in this study may be specific to our institution, it is likely that several findings are more generalizable. Future studies are needed to confirm or extend the findings from this study. Also, measures are needed for appropriate implementation of the new guidelines. These steps will likely reduce platelet usage and, in some cases, will also improve patient safety and outcomes.

## CONFLICT OF INTEREST STATEMENT

CSCohn is Chief Medical Officer for the AABB and serves on the Medical Advisory Boards for Fresenius Kabi and CellPhire Therapeutics, Inc., and on the Scientific and Research Advisory Committee for Canadian Blood Services, and as a key opinion leader for Grifols. No other conflict of interest.

## Data Availability

The data that support the findings of this study are available from the corresponding author upon reasonable request.
